# Two types of immune infiltrating cells and six hub genes can predict the occurrence of myasthenia gravis in patients with thymoma

**DOI:** 10.1080/21655979.2021.1958634

**Published:** 2021-10-08

**Authors:** Ziyou Tao, Chao Lu, Shuai Gao, Peng Zhang, Yuan Chen, Yuanguo Wang, Zhaoyu Yang, Kai Xiong, Yuxin Liu, Peng Zhang

**Affiliations:** Cardiovascular Thoracic Surgery Department, Tianjin Medical University General Hospital, Tianjin, China

**Keywords:** Thymoma, myasthenia gravis, infiltrated immune cells, Tfh cell, activated DC cells

## Abstract

Thymoma is the most common primary mass in anterior mediastinum. Although associated with low malignancy, it is often accompanied by myasthenia gravis resulting in poor prognosis. Due to the dual factors of tumor immune tolerance and autoimmune reaction, it is urgent to understand the immune status of MG with thymoma. In this study, RNA sequencing data were obtained from the TCGA and GEO cohorts to identify differentially expressed messenger RNAs and infiltrated immune cells. A total of 121 samples in TCGA and 43 samples in GEO were screened out. The infiltrated immune cells were identified by CIBERSORT, in which Tfh cells and activated DC cells were abnormal in thymoma patients. The differently expressed genes were performed by package LIMMA. The functional characteristics of differently expression genes were analyzed by GO and KEGG; one GO and seven KEGG pathways were both found in both TCGA and GEO cohorts. Meanwhile, 27 common differently expressed genes were obtained and were displayed by a Venn diagram. The TRRUST was used to screen the hub genes for the common 27 different genes and 6 genes were found. Then, PPI networks were constructed. Subsequently, the relationship between SCNAs of common genes and related immune cells tested by TIMER. Kaplan–Meier plots, ROC curve and Cox’s expression model for immune infiltration and hub genes were also tested. In conclusion, we found that two types of immune infiltrated cells and six hub genes can predict the occurrence of myasthenia gravis in thymoma patients.

## Introduction

Thymoma is the most common tumors originating from thymus epithelial cells. Thymoma patients are always associated with autoimmune diseases due to the complexity of pathological types and the effects of T lymphocyte development, of which myasthenia gravis (MG) is the most common [1]. Although the mechanism of thymoma inducing autoimmune diseases is still unclear, thymectomy for MG with abnormal thymus can effectively improve the symptoms of autoimmune diseases [2–4], which indicates the internal relationship between thymus and autoimmune diseases. The transmission dysfunction at neuromuscular junction is the main cause of MG [5]. The distinguishable symptom of MG is the aggravation of skeletal muscle weakness and fatigue, which is aggravated after activity and relieved after rest. Proteins at the neuromuscular junction are impaired due to autoantibodies, among them the most common is antibody against acetylcholine receptor (AchR) [6]. The destruction of neuromuscular junction-associated proteins is a factor in the development of MG, and the destruction of proteins is related to immune cells. Therefore, the symptom of MG is affected by immune cells. The way of abnormal thymus affecting immune cells development in MG patients remains unclear.

Immunohistochemistry and immunofluorescence are traditionally applied in the detection of tumor immune cell infiltration. However, limitation exists in terms of multiple markers stained in the same tissue section. CIBERSORT was used to analyze infiltrated immune cells in abnormal thymus by deconvolution [7]. The immune cell landscape for breast, lung and liver cancers has been successfully validated by CIBERSORT [8].

There were a lot of transcriptome dates of abnormal thymus with or without MG in TCGA and GEO cohorts. Then, we detected the immune cells in abnormal thymus. To clarify the causes of MG in thymoma, the differently expressed genes in TCGA and GEO were further analyzed. KEGG and GO analysis were used to determine the function enrichment. Furthermore, the relationship between somatic copy number alterations of common different genes and immune cells was tested in TIMER. Kaplan–Meier plots, ROC curve and Cox’s expression model for immune infiltration and hub genes were also tested. Our combined explore of immune cells and different genes confirms the effect of the immune response on the occurrence of MG in abnormal thymus.

In this study, we obtained two types of MG related immune cells and six hub genes by analyzing the immune cell infiltration and differential gene expression in thymoma microenvironment. These immune cells and hub genes were closely related to thymoma induced MG and have a certain predictive role in the development. Our results help to predict the occurrence of MG in thymoma patients, and provide new insight for the related research and clinical individualized treatment of thymoma.

## Materials and methods

### Dates from TCGA and GEO cohorts

The transcriptome expression profiles of thymoma patients were obtained from TCGA database and related clinical information was also downloaded. We obtained 87 thymoma tissues and 34 thymoma with MG tissues by 2020. Meanwhile, the transcriptome expression profiles were also downloaded from GEO database. GSE7905 with Human Genome Survey Microarray Version 2 and GPL2986 platform, and GSE29695 with Illumina HumanRef-8 WG-DASL v3.0 and GPL8432 platform were selected. There were 6 normal thymus samples and 37 thymoma samples.

### Differently expressed mRNAs in TCGA and GEO cohorts

Package LIMMA was used to screen for differentially expressed genes and fold change >2 and *P* < 0.05 were the screening conditions [9]. The different genes were showen by a Venn diagram [10].

### Evaluation of the infiltrated immune cells

CIBERSORT is an analytical tool designed by Bindea Getal, which is used to calculate the percentage of different immune cell composition fractions accurately. They included 547 genes and calculated 22 immune cells through the gene feature matrix [11]. We tested the correlation coefficient, root mean square and *P*-value for each sample and it was selected for the next analysis using only date with a *P*-value of <0.05. Finally, we obtained 78 valid samples from TCGA dataset and 40 valid samples from GEO dataset for further analysis.

## TRRUST

Transcriptional regulatory networks were constructed by TRRUST (version 2). This is an effective instrument to search for the hub genes [12].

### Enrichment analysis

We got the differently expressed genes by LIMMA package [9]. KEGG signaling pathways and GO categories were coordinated by the packages ClusterProfiler, Pathview, Colorspace, DOSE, and Stringi [13].

## TIMER

TIMER is an effective online tool for studying cancers and immune cells [14]. We used the Gene Module and SCNA Module and Survival Module to analyze the relationship between hub genes and immune cells in thymoma patients.

### PPI network

The PPI network of common differently expressed genes and hub genes was retrieved from the online tool Genemania (http://genemania.org/) [15].

### Results

Thymoma related autoimmune diseases are still a complex problem. Early prediction is of great significance for individualized treatment of patients. At present study, we committed to looking for predictors of thymoma associated MG, and we found two types of immune cells and six hub genes, and TIMER was used to identify the relation between hub genes and immune status. In additional, we performed PPI network, ROC curve and COX’s expression model to explore the prediction of the development of MG in thymoma.

### The distribution of tumor infiltration of immune cells in thymoma patients with or without MG in the TCGA cohort

**Figure 1.**
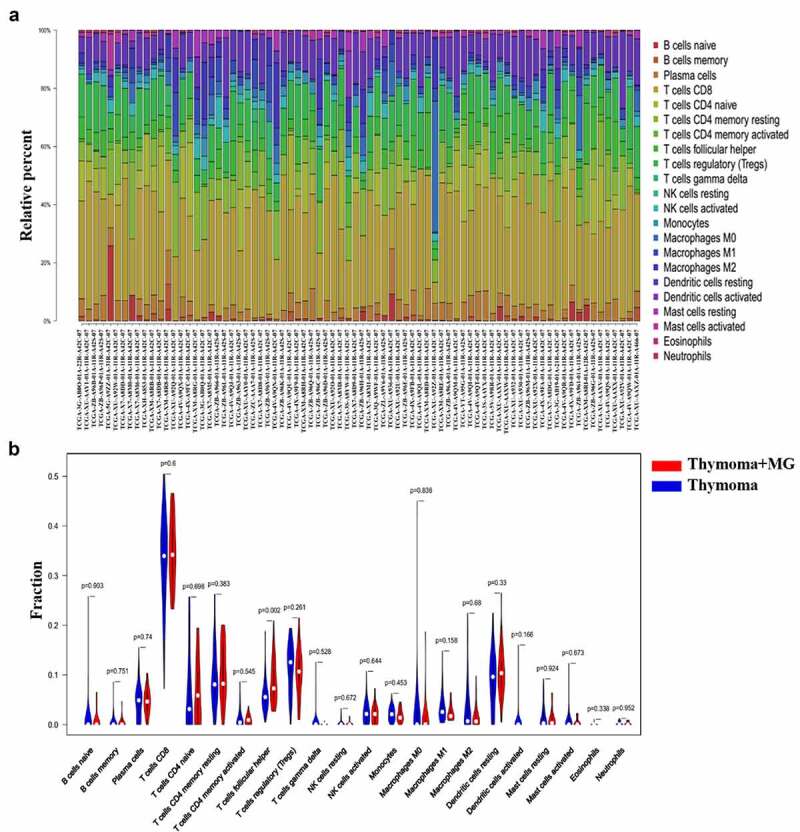
Composition of infiltrated immune cells in TCGA cohorts with CIBERSORT. (a) Fractions of immune cells in 53 thymoma tissues and 25 thymoma with MG tissues in TCGA. (b) Comparisons of immune cells in thymoma with or without MG in TCGA

Graphical abstract 1 demonstrates the process of this study. We downloaded 121 patients diagnosed with thymoma from TCGA database. The differences between 22 infiltrated immune cells in thymoma with or without MG were tested using the CIBERSORT algorithm. Among 87 thymoma without MG and 34 thymoma with MG samples, 53 thymoma without MG and 25 thymoma with MG samples were eventually selected for analysis by using the CIBERSORT (*P* < 0.05). The histograms of immune cells in TCGA are shown in Figure 1a. The level of CD8^+^T cells was the highest, accounting for about one-third of all cells. The expression of Treg cells and memory CD4^+^T cells were also high, which was consistent with the expression of resting dendritic cells, naïve CD4^+^ T cells, and plasma cells. The level of eosinophils was the low and the same for NK and activated dendritic cells. Compared with thymoma patients, Tfh cells and CD8^+^T cells increased in thymoma with MG patients, while M0, M1, M2 and Treg cells decreased. Meanwhile, only the expression of Tfh cells were significantly different (*P* = 0.002). Eosinophils and activated dendritic cells were both expressed in part of thymoma samples, but not in thymoma with MG samples (Figure 1b). Although there was no significant difference, the rare expression in thymoma with MG samples may be a clue to MG (Supplementary immune cell expression). Therefore, Tfh cells, activated dendritic cells and eosinophils may be related to the occurrence of MG in thymoma patients.

### The immune cells in thymoma tissues in the GEO datasets and the correlation among different infiltrated immune cells

**Figure 2.**
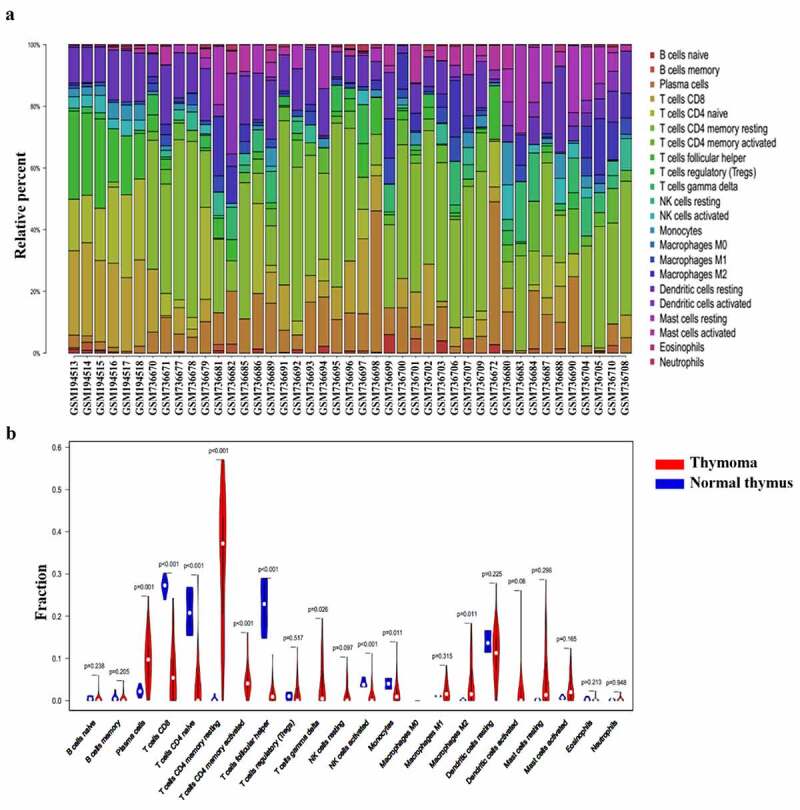
Composition of infiltrated immune cells in GEO cohorts with CIBERSORT. (a) Fractions of immune cells in six normal thymus and thymoma tissues in GEO. (b) Comparisons of immune cells in normal thymus and thymoma in GEO

GSE7905 and GSE29695 were selected to detect the immune cell in thymoma tissues. Among the 37 thymoma and 6 normal samples, 34 thymoma and 6 normal samples were eventually selected for analysis by using CIBERSORT (*P*< 0.05). In accordance with the results of TCGA, the percentage of CD8^+^ T cells and plasma cells was high, while the percentage of eosinophils, neutrophils and memory B cells was low (Figure 2a). Compared with the normal samples, the expression of resting or activated T cells and γδ T cell tended to increase in thymoma, and the same for plasma B cells, activated DC, and M2 cells. The expression of Tfh, CD8^+^ T cells, naïve CD4^+^T cells, monocytes and activated NK cells decreased in thymoma tissues. Meanwhile, all these cells were significantly different in normal thymus and thymoma tissues. Therefore, the corresponding abnormalities of T cells, B cells, monocyte, macrophages, NK cells and DC cells are associated with the development of thymoma (Figure 2b). Combined with the results of TCGA and GEO, we believed that Tfh cells and activated DC cells were involved in thymoma induced MG.

**Figure 3.**
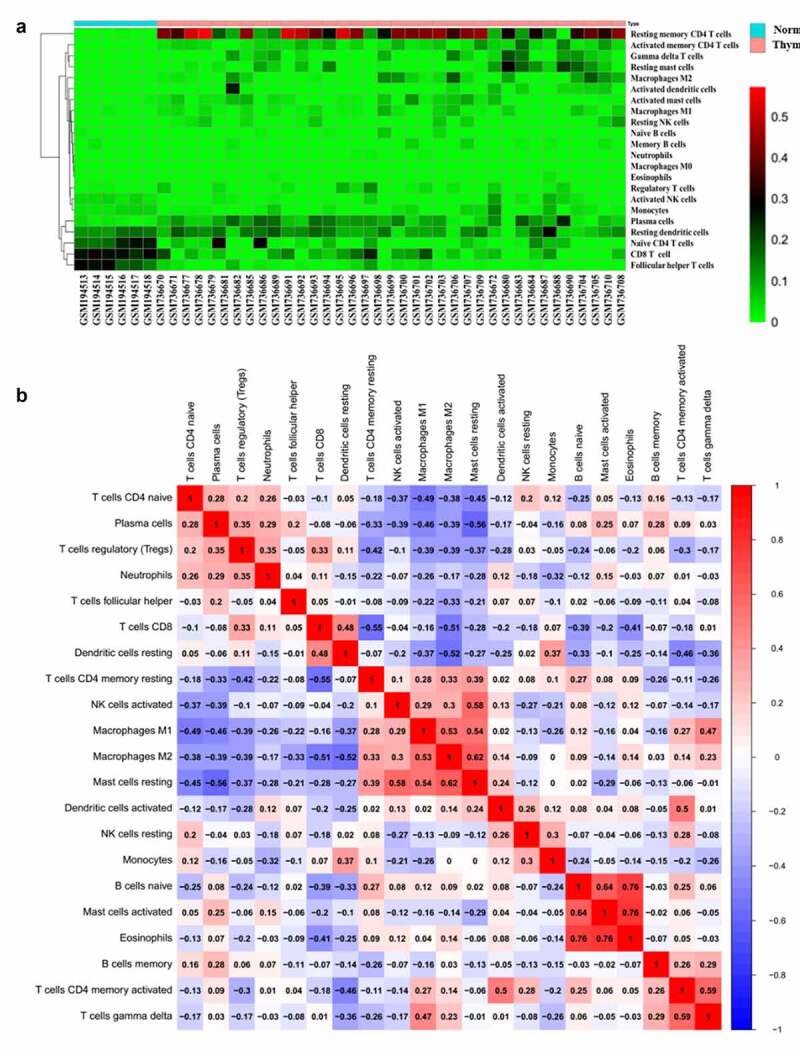
Heat map of the 22 immune cells proportions in GEO cohorts (a). Immune cells correlation diagram in GEO cohorts (b)

The heatmap of the 22 immune cells proportions in GEO (Figure 3a) and TCGA cohorts (supplementary figure 1a). The co-heatmaps were used to show the correlation among different infiltrated immune cells. Red and blue respectively represent the correlation between different cells, red represents the positive correlation between the two groups of cells and blue represents the negative correlation. The intensity of the color represents the degree of correlation. In TCGA cohort, there was a strong positive correlation between activated mast cells and eosinophils cells (R = 0.76). While, there was a negative correlation between resting mast cells and plasma B cells (R = 0.56) (supplementary figure 1b). In GEO cohort, the positive correlation between activated mast cells and eosinophils was the strongest, R-value was 0.76, the same for naïve B cells and eosinophils. The R-value between activated mast cells and naïve B cells was 0.74 and the R-value between resting mast cells and M2 cells was 0.62, there was a strong positive correlation between these cells. These findings were consistent with the results of TCGA. The expression of Tfh cells was positively correlated with plasma cells (R = 0.2), which also confirmed that Tfh cells could help B cells to produce antibodies. Activated DC cells negatively correlated with Treg cells, while the relationship with memory CD4 ^+^T cells was opposite (Figure 3b). Therefore, due to the complexity of the relationship between immune cells, further attention is needed.

### The differently expressed genes visualization and functional enrichment analysis in TCGA cohort and GEO cohort

**Figure 4.**
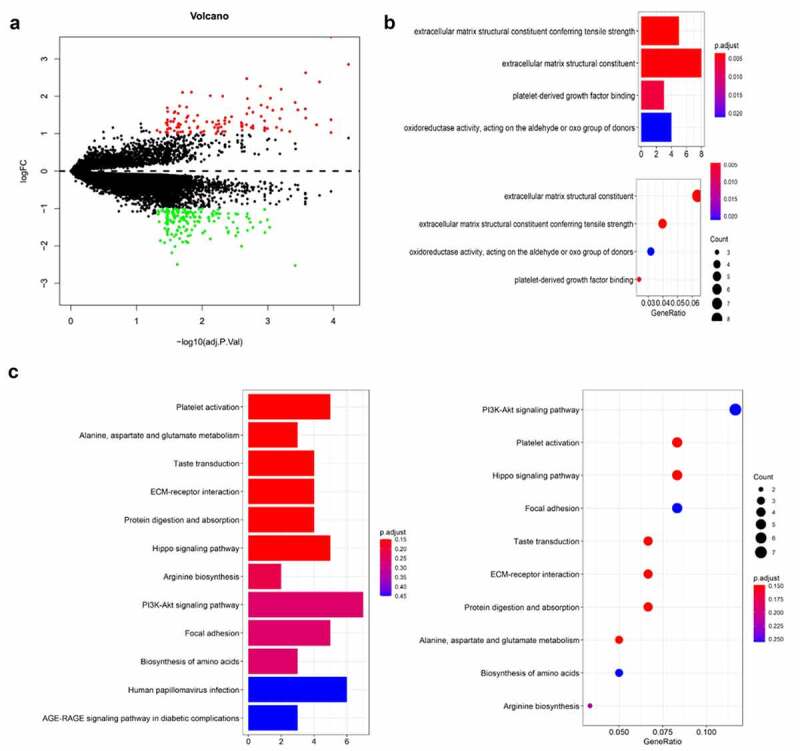
The differently expressed genes are shown by volcano map in TCGA (a). The KEGG and GO analysis are shown by bar and dot plot (b,c)

**Figure 5.**
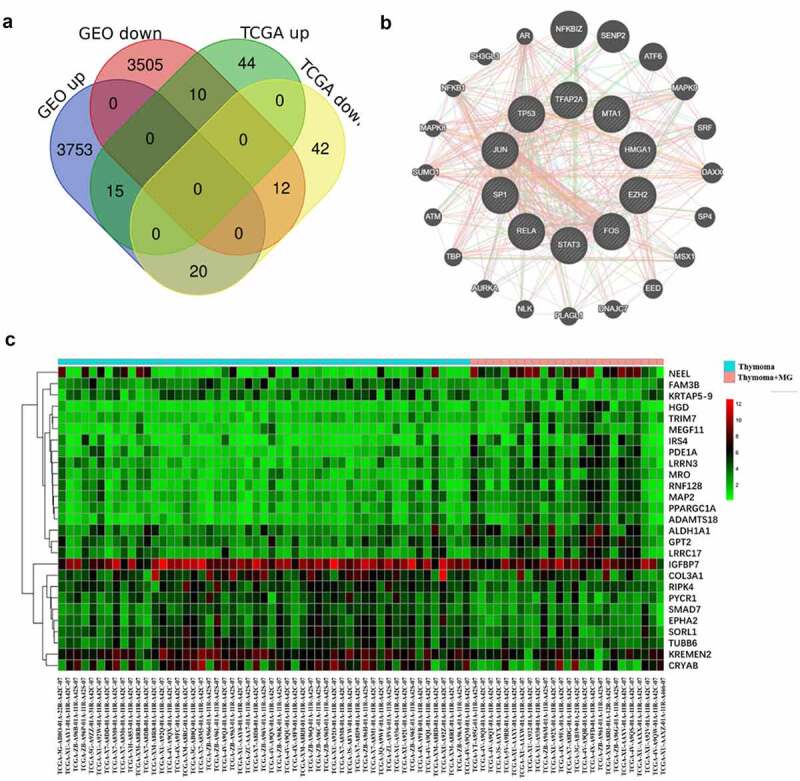
The common differently expressed genes are shown by a Venn diagram in TCGA and GEO cohorts (a). PPI network was constructed for hub genes (b). The heatmap showed the common different genes in TCGA cohort (c)

To further clarify the immune status of thymoma, the different genes in TCGA and GEO cohorts were analyzed. There were 69 upregulated genes and 74 down regulated genes, which were found after analyzing 19,347 genes in TCGA and GEO cohort by LIMMA (Figure 4a). Then, GO and KEGG enalysis were carried out on different genes (Figure 4b&4c). Using the same method, 12,589 genes in GEO cohort were analyzed, 3788 upregulated genes and 3527 downregulated genes were obtained (supplementary figure 2a). Different genes were analyzed by GO and KEGG (supplementary figure 2b&c). We found 15 upregulated genes and 12 downregulated genes in TCGA and GEO through the Venn diagram (Figure 5a). GO and KEGG analysis contains one repeated GO analysis (GO:0005201) and seven repeated signaling pathways (hsa04611, hsa04390, hsa04151, hsa04510, hsa05165, hsa04933 and hsa0430).

### Hub genes in TCGA and GEO cohorts and interaction analyses

TRRUST was used to screen the common 27 different genes. Ten hub genes were found, but only six genes were statistically different (*P* < 0.05). These six hub genes are *HMGA1*, *MTA1*, *EZH2*, *FOS*, *TP53*, and *TFAP2A*. The heatmap was used to show the common different genes in TCGA (supplementary figure 3a) and GEO cohorts (Figure 5c).

The online tool Genemania was used to construct PPI networks of hub genes and common different genes. The results of PPI prediction of common different genes showed that co-expression accounted for 61.66%, physical interactions accounted for 21.32%, predicted accounted for 11.30%, and co-localization accounted for 5.72%. The results of hub genes showed that physical interactions accounted for 51.71%, co-localization accounted for 16.48%, genetic interactions accounted for 15.26%, predicted accounted for 14.12%, shared protein domains accounted for 1.93% and the pathway accounted for 0.5% (Figure 5b and supplementary figure 3b). Therefore, there were extensive interactions between hub genes and different common genes.

### The connection between hub genes and immune cells in thymoma

**Figure 6.**
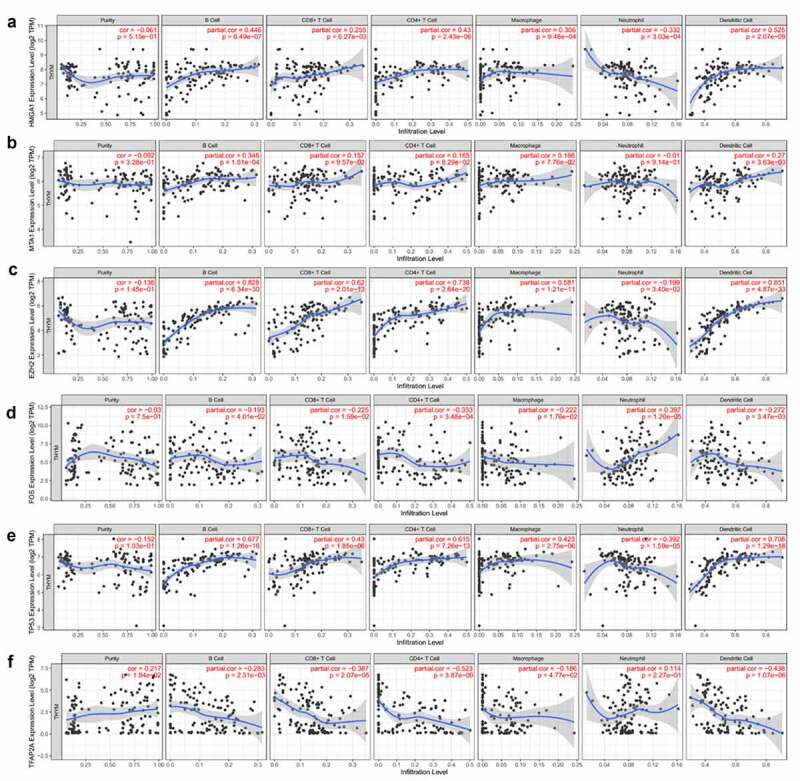
The relationship between genes of *HMGA1* (a), *MTA1* (b), *EZH2* (c), *FOS* (d), *TP53* (e), *TFAP2A* (f) and immune cells infiltration was verified in TIMER

The connection between gene expression and infiltrated immune cells was tested in TIMER. In the gene module of TIMER, we found that genes *HMGA1*, *EZH2*, *TP53*, and *MTA1* were positively corelated with CD4^+^T cells, the same for CD8^+^T cells, Macrophage cells and DC cells. Meanwhile, the genes (*HMGA1*, *EZH2* and *TP53*) were negatively correlated with neutrophil cells. For *FOS* and *TFAP2A* gene analysis, the results showed that there was a negative correlation between these genes and CD4^+^T cells, the same for CD8^+^T cells, macrophage cells, and DC cells, while the expression of *FOS* was positive with neutrophil cells (Figure 6).

**Figure 7.**
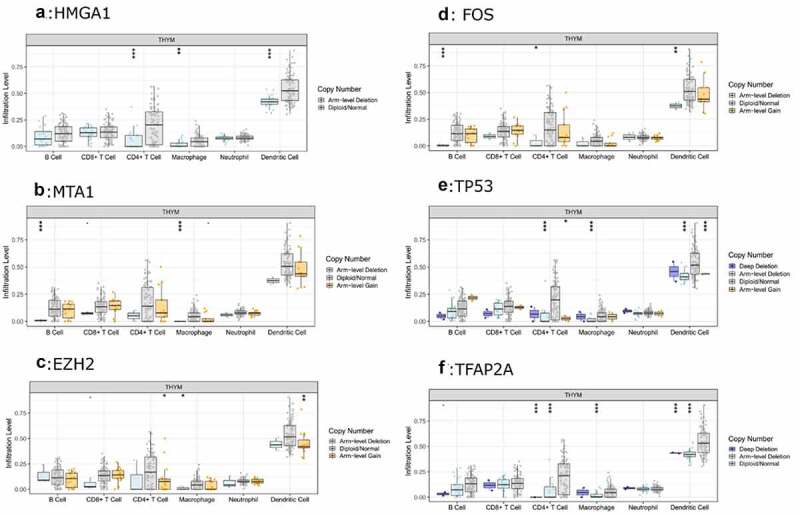
The relationship between the SCNA changes of *HMGA1* (a), *MTA1* (b), *EZH2* (c), *FOS* (d), *TP53* (e), *TFAP2A* (f) and the expression level of immune cells was verified by TIMER

**Figure 8.**
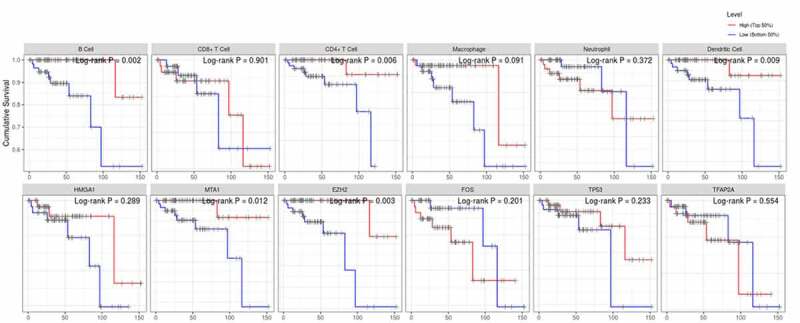
The survival differences of hub genes and immune cells were tested by KM plots

**Figure 9.**
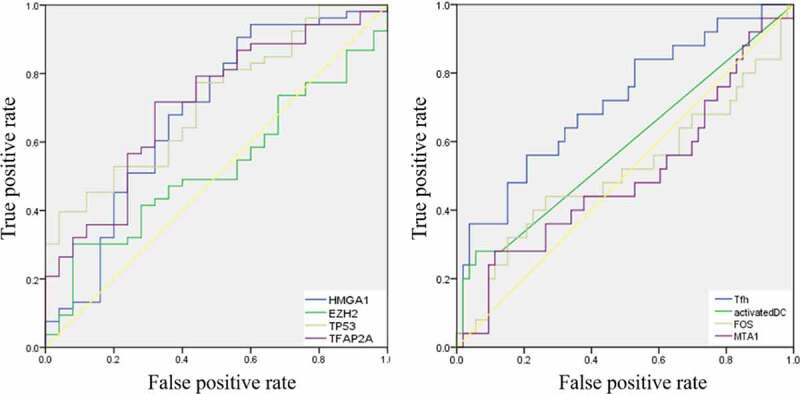
ROC curves for validation the occurrence of MG in thymoma patients

Then, the comparison of immune cells and the SCNA changes of hub genes was tested in TIMER and we use GISTIC 2.0 to define the SCNAs. The distribution of immune subsets in each copy number state is shown by the boxed graph. The SCNA changes of genes *HMGA1*, *TP53*, *EZH2*, and *TFAP2A* were related to CD4^+^T cells, Macrophage cells, and DC cells, while the SCNA change of genes *FOS* and *MTA1* were related to B cells (Figure 7). Meanwhile, the changes of *FOS* werealso related to DC infiltration and the changes of *MTA1* were related to macrophage cells. At last, we used TIMER to plot Kaplan–Meier plot of immune infiltration and hub genes to show survival differences. By sliding the user-defined slider to 50%, it was divided into low-level and high-level. Finally, difference among B, T and DC cells was statistically significant, which were also found in *MTA1* and *EZH2* genes. Longer survival period was related to higher expression of B cells and dendritic cells, and we also found the same results in CD4^+^T cells, *MTA1* and *EZH2* (Figure 8). The COX model of six hub genes and immune cells in thymoma patients (Table 2). ROC was tested for validation in prediction of the occurrence of MG and the results showed that Tfh cells and genes such as *HMGA1*, *TP53* and *TFAP2A* could effectively predict MG occurrence in thymoma patients (Figure 9 and Table 1). In conclusion, these genes affect the expression and function of immune cells, thus predicting the occurrence of MG in patients with thymoma.

**Table 1.** ROC analysis of the immune cells and hub genes

	AUC	*P*-value	95% CI
Tfh	0.715	**0.02**	0.591–0.840
Activated DC	0.586	0.224	0.443–0.729
*FOS*	0.511	0.877	0.361–0.661
*MTA1*	0.499	0.987	0.354–0.643
*HMGA1*	0.683	**0.009**	0.547–0.819
*EZH2*	0.526	0.712	0.395–0.657
*TP53*	0.718	**0.002**	0.602–0.834
*TFAP2A*	0.709	**0.003**	0.587–0.831

Tfh, follicular helper T cells; DC, dendritic cells; AUC, area under curve; CI, confidence interval.

**Table 2.** We established a COX model of six hub genes and immune cells in patients with thymoma

	Coef	HR	*P*-value	95% CI
Age	0.182	1.2	**0.032**	1.016–1.417
Gender	−0.644	0.525	0.589	0.051–5.438
Purity	1.787	5.973	0.513	0.028–1266.668
B cell	−56.649	0	**0.029**	0–0.003
CD8 T cell	−14.822	0	0.447	0–1.41E+10
CD4 T cell	−44.501	0	0.089	0–876.375
Macrophage	−16.213	0	0.401	0–2.53E+09
Neutrophil	−80.659	0	0.114	0–2.89E+08
Dendritic	47.627	4.83E+20	0.145	0–3.31E+48
*HMGA1*	−2.364	0.094	0.101	0.006–1.585
*MTA1*	2.38	10.803	0.214	0.254–459.641
*EZH2*	1.579	4.85	0.38	0.143–164.672
*FOS*	0.558	1.747	0.078	0.939–3.253
*TFAP2A*	−1.917	0.147	**0.044**	0.023–0.954
*TP53*	2.104	8.197	0.243	0.24–280.172

HR, hisk risk; CI, confidence interval; Coef, coefficient.

## Discussion

At present, researches of immune cell infiltration and immunotherapy mainly focus on malignant tumors, and few studies put emphasis on thymoma. Immunotherapy targeting PD1 and PDL1 can reverse immune tolerance and demonstrate significant clinical efficacy in malignant tumors, but the therapeutic effect of targeting PD1 and PDL1 in thymoma is not so well. The immune-targeted therapy in tumors reverses the immune tolerance and enhances the immune response, which is in turn a risk factor for autoimmune diseases. In thymoma with MG patients, tumor immune tolerance combined with autoimmune status simultaneously exist, which may account for poor effect of tumor immunotherapy. Therefore, understanding the immune status and immune cell infiltration of thymoma with MG is of great significance for further understanding the progress of MG. Compared with normal thymus samples, there were extensive abnormal expression of immune cells in thymoma samples, especially for T lymphocytes, and the same for B cells, NK cells, dendritic cells and macrophages. Thymus, as a central immune organ, is the place of T lymphocyte differentiation and development. The pathological changes of thymus will inevitably affect the changes of T lymphocyte subsets, and also affect other immune cells to a certain extent. The difference of Tfh cells was only found in thymoma with or with out MG samples. Activated dendritic cells were rarely expressed in thymoma with MG samples, which may be related to MG. The little difference between two groups confirmed that thymoma induced MG may be more insidious, and more detailed lymphocyte subsets need to be paid more attention. Previous studies showed that the expression of tumor-associated Tfh infiltration increased in the thymoma microenvironment [16–18]. Tfh cells could interact with B cells to help B cells produce antibodies in patients with thymic hyperplasia, which is one of the factors inducing MG [19]. Patients with early improvement after thymectomy, memory lymphocytes and mature DC cells can be seen around the blood vessels, the author believed that thymectomy may block the production of mature DC cells and the output of activated T cells, so as to improve the disease status [20]. At the same time, other researchers found that CD8^+^T cells stimulated by IL-6 transfected DC cells have strong cytoxicity and survival advantages, which suggests that DC cells in different states may affect the number and function of immune cells, thus affecting the progression of the disease [21].

In the second part of the study, we found the destruction of the ECM structure might affect the progression of MG. Extracellular matrix (ECM) actively participates in the control of cell differentiation. It is involved in maintaining cell structure, regulating homeostasis, and affecting the interaction between cells and molecules through specific receptors. Although people know little about the composition and function of ECM, the expression of ECM components has changed significantly in a variety of neurological diseases, including multiple sclerosis, osteoarthritis [22] and rheumatoid arthritis [23]. Therefore, the destruction of the ECM structure may be a cause of thymoma related MG. PI3K-AKT signaling pathway controls the differentiation of immune cells. In mice, the differentiation of T cells into Th cell lineage can be inhibited by the PI3K-AKT signaling pathway. While in humans, PI3K deficiency can cause autoimmune liver inflammation, significantly reduce B lymphocytes and memory T cells [24]. MST1/2, a signaling molecule of Hippo signaling pathway, is an immunosuppressive molecule, which can regulate the adhesion and migration of lymphocytes, inhibit the adaptive immune response by regulating the development of initial T cells and regulatory T cells [25]. It has been found that RAGE interacts with HMGB1 to activate dendritic cells and B cells, which may lead to autoimmune diseases by changing antigen presentation to T cells and other suitable effector cells [26]. Other researches showed that RAGE has a direct impact on the mobilization of dendritic cells and also has some effects on the migration, localization, activation, and differentiation of T cells. Therefore, the function of immune cells in thymoma are regulated by these signaling pathways.

It has been found that HMGA1 is expressed in thymus tissue, and its upregulation can affect the competition process of T cell precursor cells in thymus, leading to abnormal differentiation and maturation of T cells. In HMGA1 knockout mice model, the number of T cell precursor cells decreased and B cells differentiated preferentially. Compared with early thymoma, advanced thymoma has higher MTA1 levels. Therefore, these researchers firmly confirmed that the aggressiveness of thymoma was regulated by the expression of *MTA1* gene. Meanwhile, MTA1 contains CD4 T cell epitopes that can promote the secretion of IFN-γ, and is involved in the development of mouse thymocytes under activated conditions [27]. It has been proved that EZH2 deletion is associated with the disorder of Treg differentiation in vitro [28]. Fra1 is upregulated in MG than that in normal thymus, and researchers have confirmed the overexpression of Fra1 in medulla thymic epithelial cells, which destroys the secretion of inflammatory cytokines and affects disease progression [29]. MiR-205-5p negatively control thymic involution by interacting with *TFAP2A* [30]. There was a strong correlation between these genes and immune status of thymus, while there was little research on thymoma induced MG which should be paid more attention.

In order to figure out the relationship between six hub genes and immune cell infiltration in thymoma, TIMER was used to verify this issue. *HMGA*1, *EZH2*, *TP53*, and *MTA1* were significantly related to T cells, indicating their effects on T cell differentiation and development. There was also an obvious relationship between neutrophil cells and hub genes, which may be the driving factor of MG. The SCNA changes of genes *HMGA1*, *TP53*, *EZH2*, and *TFAP2A* were related to CD4^+^T cells, macrophage cells, and DC cells, and the SCNA changes of genes *FOS* and *MTA1* were related to B cells. At the same time, *FOS* was also related to DC infiltration and MTA1 was related to macrophage cells. The change of SCNA in hub genes further explained the internal relationship between hub genes and immune cell infiltration. All these studies indicate that these genes are related to the invasion of immune cells in thymoma and are involved in the occurrence of MG.

There are some shortcomings about this article. First, because of limited researches on thymoma, only two databases in the GEO cohort met the purpose requirements. Second, due to thymus degeneration in adults, there were only few normal tissue samples in TCGA cohort. Third, the immune cells and hub genes obtained in this study have not been verified by experiments.

## Conclusion

We detected the levels of 22 immune cells in thymoma microenvironment and studied their roles in the occurrence of MG. We found that the expression of Tfh cells and activated DC cells are both abnormal in thymoma with MG patients. Six hub genes were obtained by analysis of different genes. The relationships between the expression and somatic copy number alterations of hub genes and infiltrated immune cells were tested in TIMER, Kaplan–Meier plots, ROC curve, Cox’s expression model for immune infiltration and hub genes were also tested. All these data indicated that hub genes are involved in the occurrence of MG in thymoma patients. We hope these results can provide novel point in the causes of MG, thus helping clinicians in evaluating the diagnosis, treatment, and then improving the prognosis of thymoma with MG.

## Supplementary Material

Supplemental Material

## Data Availability

All data can be obtained from TCGA and GEO cohorts.

## Highlights

1. The infiltration of Tfh cells and activated DC cells is abnormal in thymoma patients.

2. Six hub genes were obtained by analysis of thymoma patient’s data from TCGA and GEO cohorts.

3. The infiltration of Tfh cells, activated DC cells and six hub genes are predictors of the occurrence of MG in thymoma patients.
