# Supplementary material for: Two types of immune infiltrating cells and six hub genes can predict the occurrence of myasthenia gravis in patients with thymoma
Source: Bioengineered. 2021 Oct 8;12(1):5004–16. doi: 10.1080/21655979.2021.1958634 (PMC8806799; doi:10.1080/21655979.2021.1958634)
Supplement: Supplemental Material [file KBIE_A_1958634_SM2068.zip › Supplementary figures_revised.docx]

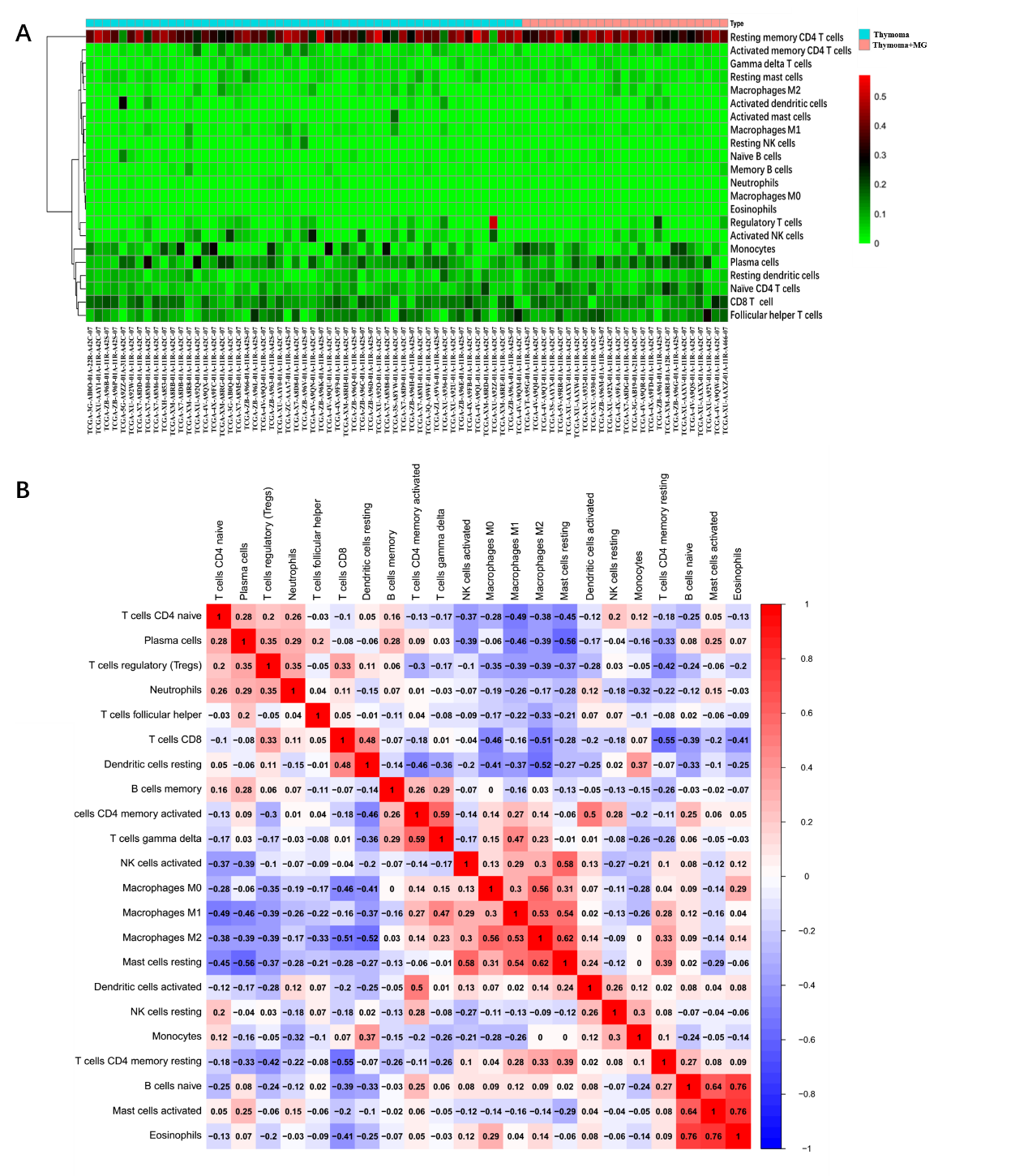


Supplementary figure1. The landscape of infiltrated immune cells in thymoma. Heatmap of the 22 immune cells proportions in TCGA cohort (A). Correlation matrix of 22 immune cells densities in the TCGA and GEO cohort (B).


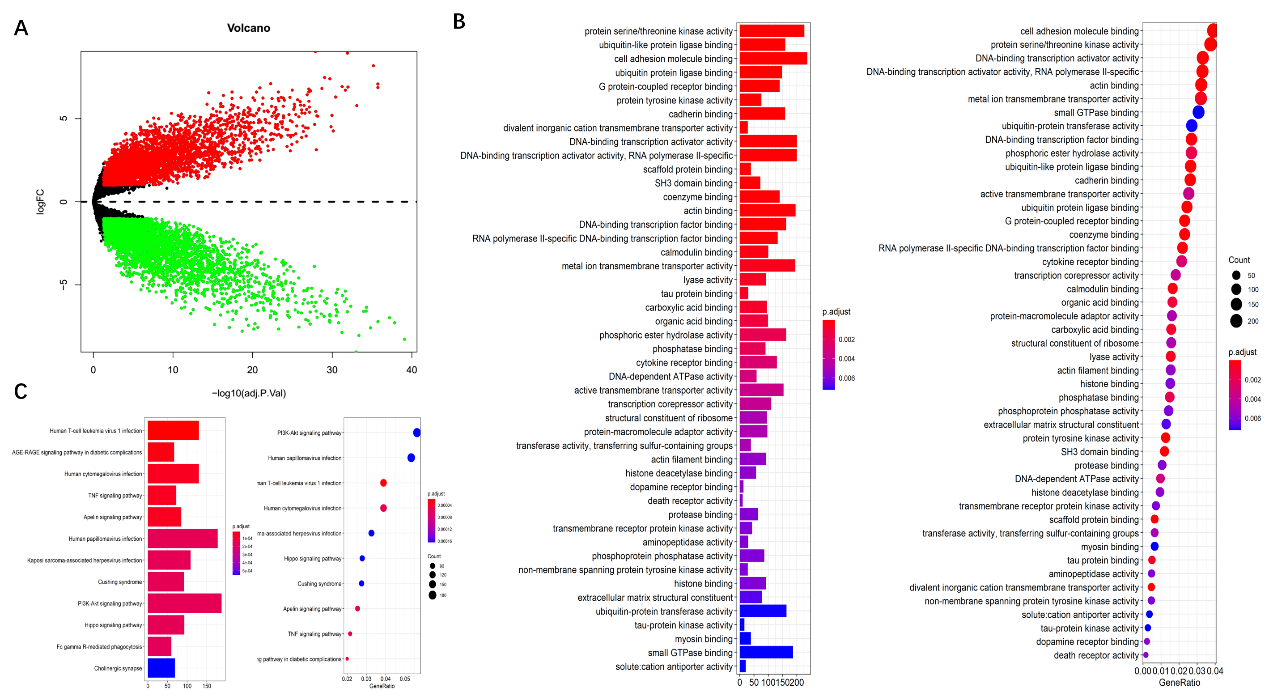


Supplementary figure2. Differentially expressed genes in GEO cohort and the enrichment analysis of differentially expression genes. The volcano map showed the differentially expression genes in GEO (A). Bar and dot plot showing the KEGG pathway and GO analysis in GEO cohort (B, C). The correlation is more significant as the red/blue ratio increases.


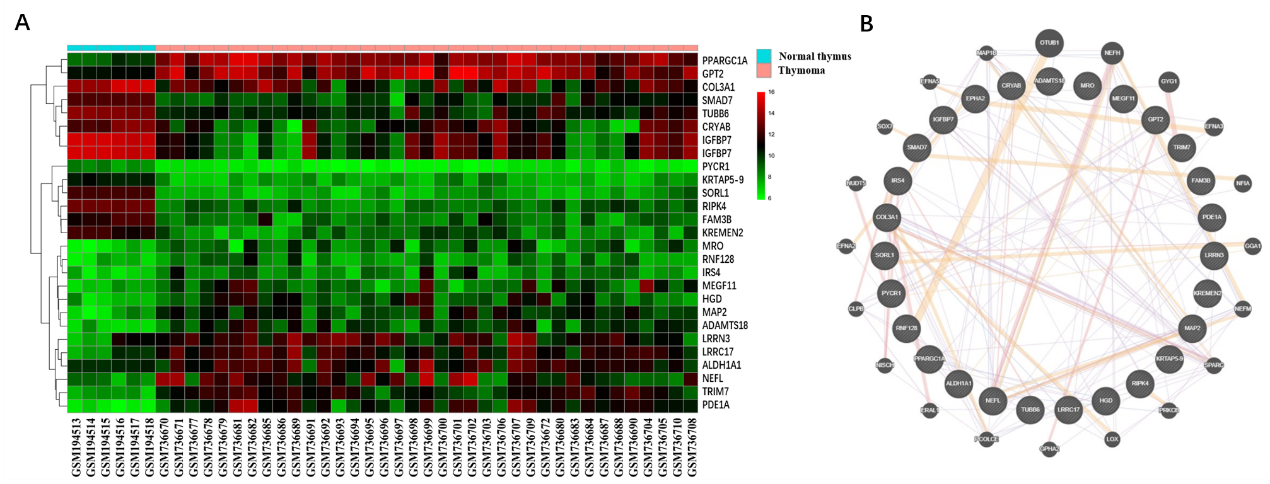


Supplementary figure3. The common differentially expression genes in GEO cohort and the protein-protein interaction network. The heatmap showed the common differential genes in GEO cohort (A). The protein-protein interaction network of common differentially expression genes (B).
